# Increased depression and metabolic risk in postmenopausal breast cancer survivors

**DOI:** 10.1186/s13098-016-0170-4

**Published:** 2016-07-22

**Authors:** Monica C. Serra, Andrew P. Goldberg, Alice S. Ryan

**Affiliations:** Division of Gerontology and Geriatric Medicine, Department of Medicine, University of Maryland School of Medicine and Geriatric Research and Education Clinical Center, Baltimore VA Medical Center, 10 N Greene St. (BT/18/GR), Baltimore, MD 21201 USA

## Abstract

**Objective:**

Breast cancer survivors (BCS) are at high risk for the development of obesity, type 2 diabetes mellitus, and metabolic syndrome. There is increasing interest in the association between depression and metabolic dysfunction, which is relevant in this population as depression is often present in the chronic phase of cancer recovery. Thus, the aim of this study was to evaluate metabolic risk in BCS with and without depression compared to non-cancer controls.

**Methods:**

African American (46 %) and Caucasian (54 %) postmenopausal BCS (N = 28; age: 60 ± 2 years; mean ± SEM) were matched for race, age (±2 years), and BMI (±2 kg/m^2^) to non-cancer controls (N = 28). Center for Epidemiologic Studies Depression Scale (CES-D) >16 or antidepressant medication usage was used to classify depression. Metabolic status was defined by 2-hr glucose during an OGTT and classification of metabolic syndrome.

**Results:**

Compared to non-cancer controls, BCS had similar 2-hr glucose, but higher fasting glucose and total cholesterol, and were 2.5 times more likely to have metabolic syndrome (21 vs. 52 %)(P’s < 0.05). Conversely, HDL-C was 16 % higher in BCS (P < 0.05). Forty three % of BCS were on antidepressants compared to 14 % in non-cancer controls, despite similar mean CES-D scores (6 ± 1). Depressed BCS (46 %) had a higher BMI, waist circumference, fasting glucose, and more metabolic syndrome components than non-depressed BCS (P’s < 0.05).

**Conclusions:**

BCS have a heightened prevalence of depression that may be associated with an increased prevalence of metabolic syndrome. These results support the need to monitor weight gain, depression, and the progression of metabolic abnormalities after cancer diagnosis and treatment. Further studies into the mechanistic link between depression and metabolic disease are necessary to identify strategies that can offset their impact on obesity and associated cardiovascular risk following a breast cancer diagnosis.

## Background

Due to better breast cancer screening modalities and cancer treatment options [1], the number of breast cancer survivors (BCS) in the United States is expected to increase from ~3 million survivors in 2012 to 3.8 million by 2022 [2]. Although more women are surviving this disease, a higher prevalence of depressive symptoms exists among BCS compared to the general female population, often persisting more than 5 years after diagnosis [3]. BCS also are at high risk for the development of obesity and associated cardiovascular disease risk, including metabolic syndrome, type 2 diabetes mellitus (T2DM), and hypertension [4, 5]. Depression and heightened metabolic dysfunction in chronic cancer BCS are often attributed to cancer diagnosis and treatment side effects, including emotional distress, pain, sleep disturbances, and excessive adiposity [6, 7].

Depression may affect the ability to make positive lifestyle changes and comply with medical therapy [8], while antidepressant treatment is associated with weight gain [9], possibly placing BCS at even greater metabolic risk. Indeed, a recent meta-analysis concluded that depression is associated with worse glycemic control, poor adherence to medication and diet regimens, and a reduction in quality of life in adults with T2DM [10]. Large waist circumference and low high-density lipoprotein cholesterol (HDL-C) also show associations with depression in subjects with an unknown cancer history [11]. Thus, determining the presence of depression in BCS may have implications for clinical care in the chronic phase of recovery.

The tendency for weight gain with aging places postmenopausal women at increased risk for metabolic dysfunction [12]. In addition to abdominal obesity, significant risk factors for breast cancer in postmenopausal women are elevated fasting glucose, hypertension, hyperlipidemia [13], all components of the metabolic syndrome. Less is known about whether cancer survivorship increases metabolic risk during long-term recovery. Only a few studies have matched postmenopausal BCS to postmenopausal non-cancer controls [4, 14, 15], finding that BCS are more likely to have metabolic abnormalities than age-matched women without prior breast cancer. However, several limitations are present in these studies, including differences in BMIs between cases and controls and/or lack of consideration of differences in racial profiles between groups, which could affect interpretability of the results. Thus, this study evaluated metabolic risk and depression in postmenopausal BCS compared to postmenopausal women without a prior diagnosis of breast cancer matched for age, race, and BMI. We hypothesized that women with previous breast cancer have worse depression scores and poorer metabolic profiles compared to women without a history of breast cancer, and that this difference is independent of age, race, and obesity. Further, we anticipate that BCS with depression will have greater metabolic dysfunction than BCS without depression.

## Methods

### Study design and sample selection

This cohort study was observational in nature. Groups were comprised of 28 postmenopausal BCS and 28 postmenopausal women without prior breast cancer diagnosis matched for race, age (±2 years), and BMI (±2 kg/m^2^). All women reported being sedentary (<30 min of structured exercise, two times per week) and weight stable (<2 kg weight change) over the prior six months. Women without a history of breast cancer were selected from a larger subset of individuals from a previously published manuscript [16]. BCS were recruited from the Baltimore area and had completed surgical treatment, radiation therapy, and/or chemotherapy at least 6 months prior to enrollment. All women signed University of Maryland Institutional Review Board approved informed consent forms. A medical history, physical examination, resting 12-lead electrocardiogram, and fasting blood profile were performed to determine current metabolic abnormalities.

### Cardiorespiratory fitness

VO_2_ was measured by indirect calorimetry during a graded exercise test on a treadmill as previously described [17]. VO_2_max was accepted as valid if two of the three following criteria were met: respiratory exchange ratio ≥1.0, maximum heart rate >90 % of age-predicted maximum (220-age), or a plateau in VO_2_ (<200 ml/min change). If such criteria were met, the highest level of VO_2_ was defined as VO_2_max.

### Depression assessment

Risk for depression was measured with the Center for Epidemiologic Studies Depression Scale (CES-D). The CES-D is composed of 20 items and assesses risk for depression in the general population. A cut off of 16 was used to indicate “mild” depressive symptomatology [18]. Subjects were classified as having depression if they had a CES-D score >16 and/or were being treated with an antidepression medication.

### Clinical parameters and laboratory procedures

Height, body weight, and waist and hip circumferences were measured using standardized protocols [19], and BMI (kg/m^2^) and waist to hip ratio (WHR) calculated. Blood pressure was measured on the non-affected cancer side for BCS and on the right side in the non-cancer controls after 10 min of resting on three occasions and averaged.

Blood was collected after a 12 h fast. Plasma triglyceride and cholesterol levels were analyzed using enzymatic methods (UniCelDxC880i; Beckman Coulter, Inc., Brea, CA), HDL-C measured in the supernatant after precipitation with dextran sulfate, and low-density lipoprotein cholesterol (LDL-C) calculated as LDL-C = total cholesterol- HDL-C -TG/5 [20]. A oral glucose tolerance test was performed, with glucose measured at fasting and every 30 min. for 2 h following the ingestion of 75 g glucose load. Glucose was measured by the glucose oxidase method (2300-STAT Plus; YSI, Yellow Springs, OH) and insulin measured by radioimmunoassay (Linco Research Inc., St. Charles, MO) [21]. The presence of three or more of the following metabolic syndrome components were used to diagnose metabolic syndrome: central obesity (waist >88 cm), impaired glucose metabolism (fasting glucose >5.6 mmol/L), elevated blood pressure (>130/85 mmHg or antihypertensive treatment), and dyslipidemia (triglyceride (TG) >1.7 mmol/L, HDL-C <1.3 mmol/L, or hypolipidemic treatment) [22].

### Statistical analyses

Data were analyzed using SPSS Version 20. Data were analyzed for normality using the Kolmogorov–Smirnov test. Mean ± SEM were calculated for continuous variables and compared using Student’s t test after log transformation of variables (plasma triglycerides), as appropriate. Percentages were calculated for categorical variables (presence vs. absence of medication usage, metabolic syndrome, and depression) and compared using χ^2^ tests. Pearson correlation coefficients were calculated to determine relationships between variables. All tests were two-tailed, and P < 0.05 were considered statistically significant.

## Results

### Subject characteristics

The women were 46 % African American and 54 % Caucasian. BCS were an average of 94 ± 18 (range 9–384) months since diagnosis. Treatment was lumpectomy in 54 % and mastectomy in 57 %. Forty-three percent underwent chemotherapy alone, 46 % radiation therapy alone, and 11 % received both. At study entry, 25 % of BCS were taking an aromatase inhibitor, 12 % tamoxifen, and 63 % were not receiving hormone therapy. BCS were well matched to non-cancer controls with regard to age, BMI, waist circumference, and WHR, but VO_2_max was 25 % higher in BCS (P < 0.05) (Table 1).

**Table 1:** Demographic characteristics of non-cancer controls and breast cancer survivors

	Control N = 28	BCS N = 28
Age (years)	60 ± 2	60 ± 2
BMI (kg/m^2^)	32 ± 1	32 ± 1
Waist circumference (cm)	96 ± 3	93 ± 1
WHR	0.81 ± 0.01	0.79 ± 0.01
VO_2_ max (ml/kg/min)	18.8 ± 1.0	23.5 ± 1.5^a^

Mean ± SEM

*BMI* body mass index; *WHR* waist to hip ratio

Different than control: ^a^ P < 0.05

### Elevated metabolic abnormalities and depression in breast cancer survivors (Table 2)

On average, BCS had slightly elevated fasting glucose, with 9 % higher fasting glucose than non-cancer controls (P < 0.05). BCS also had higher HDL cholesterol (P < 0.05), tended to have higher total cholesterol (P = 0.08), and 39 % more required a lipid lowering medication (P < 0.01). Diastolic and systolic blood pressure and hypertension medication usage was similar between groups. BCS were 2.5 times more likely to have metabolic syndrome (21 vs. 52 %; P < 0.05), with blood pressure (64 %) and waist circumference (61 %) being the most prevalent component in BCS.

**Table 2:** Elevated metabolic abnormalities and depression in breast cancer survivors

	Control N = 28	BCS N = 28
Systolic blood pressure (mmHg)	121 ± 3	122 ± 2
Diastolic blood pressure (mmHg)	68 ± 1	68 ± 1
Hypertension medication usage (%)	43	56
Fasting glucose (mmol/L)	5.2 ± 0.1	5.7 ± 0.2^b^
Fasting insulin (pmol/L)	86 ± 7	97 ± 15
HOMA-IR	3.3 ± 0.3	4.1 ± 0.6
2-hr glucose (mmol/L)	7.1 ± 0.4	6.6 ± 0.6
Cholesterol (mmol/L)	4.8 ± 0.1	5.2 ± 0.2^a^
Triglycerides (mmol/L)	1.2 ± 0.1	1.4 ± 0.1
HDL-C (mmol/L)	1.4 ± 0.1	1.7 ± 0.1^b^
LDL-C (mmol/L)	2.8 ± 0.1	2.9 ± 0.2
Lipid lowering medication usage (%)	11	50^c^
Metabolic syndrome components (#)	1.7 ± 0.2	2.4 ± 0.3^b^
CES-D	6 ± 1	6 ± 1
Antidepressant medication usage (%)	14	43^b^

Mean ± SEM

*HDL*-*C* high-density lipoprotein cholesterol; *LDL*-*C* low-density lipoprotein cholesterol; *CES*-*D* Center for Epidemiologic Studies Depression Scale

Different than control: ^a ^P = 0.08; ^b^ P < 0.05; ^c^ P < 0.01

CES-D scores >16 were observed in ~14 % of non-cancer control and BCS subjects. Despite similar mean CES-D scores, three times more BCS were being treated with an antidepressant medication (P < 0.05; 58 % of BCS were on a selective serotonin reuptake inhibitor, 25 % on an atypical antidepressant, and 17 % on a tricyclic antidepressant). Twenty-six % of non-cancer controls and 46 % of BCS were classified as depressed (CES-D >16 and/or on an antidepressant) (P = NS).

### Comparisons of metabolic abnormalities by depression status in breast cancer survivors

**Fig. 1:**
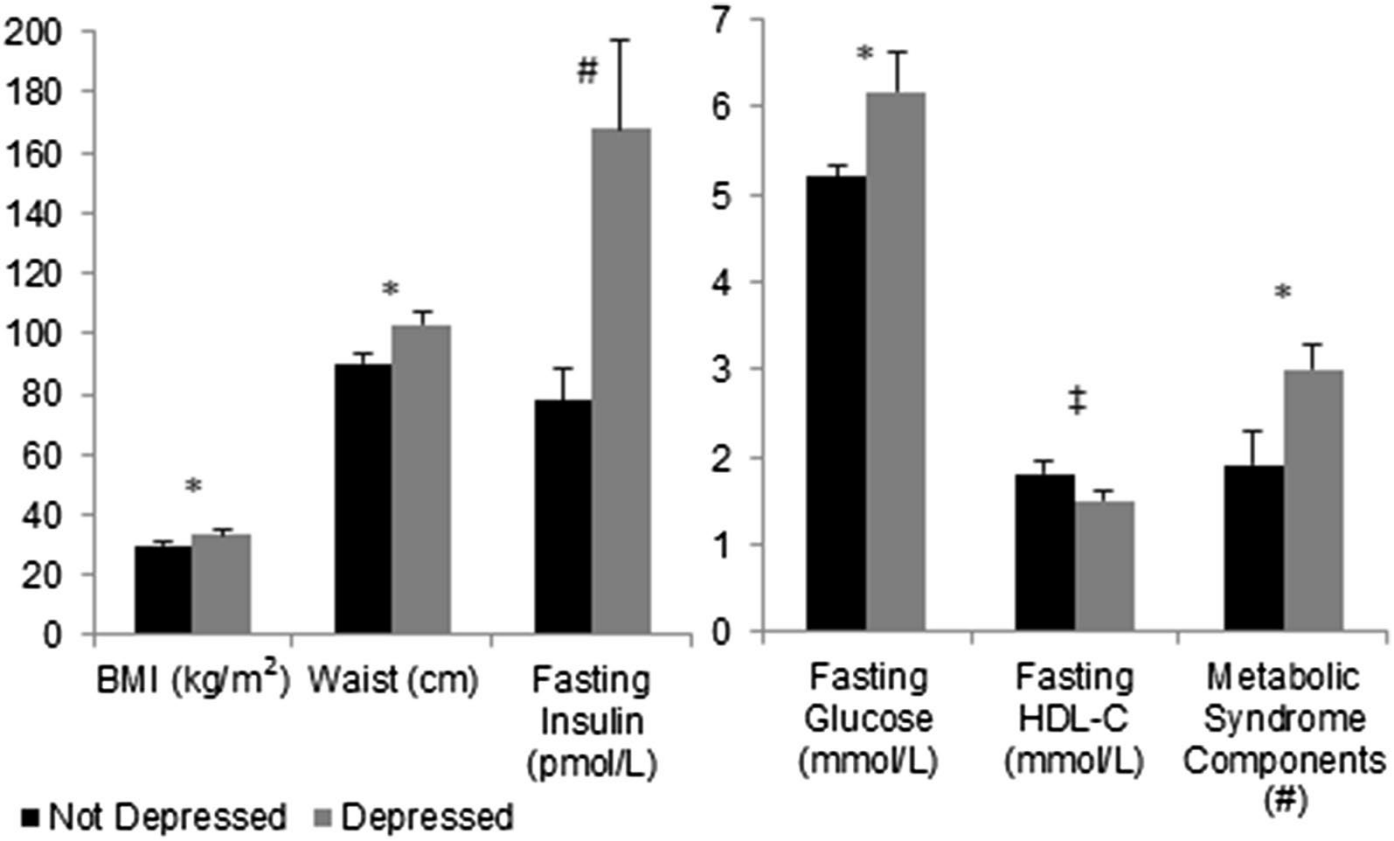
Prevalence of obesity and metabolic profiles of breast cancer survivors with (n = 13) and without (n = 15) depression (*P < 0.05, ^#^P = 0.07, ^‡^P = 0.09)

As anticipated, BCS classified with depression had higher mean CES-D scores than BCS without depression (4.1 ± 0.9 vs. 8.5 ± 2.0, P < 0.05). Despite similar age, racial profiles, fitness levels, and time from cancer diagnosis (data not shown), BCS with depression also had a higher BMI, waist circumference, fasting glucose, and more metabolic syndrome components (P’s < 0.05; Fig. 1) than non-depressed BCS. Further, there was a trend for fasting insulin to be higher (P = 0.07) and HDL-C (P = 0.09) to be lower in BCS with depression (Fig. 1). Blood pressure and all other lipid variables were similar between groups, including the usage of hypertension and lipid lowering medications (data not shown).

## Discussion

While numerous studies have identified weight gain as a consequence of breast cancer treatment extending into the chronic phase of recovery [23, 24], fewer studies have examined the prevalence of metabolic dysfunction in BCS. Although studies have reported that postmenopausal BCS have more metabolic abnormalities than non-cancer controls [4, 14], ours is the first to show that this is independent of age, race, and obesity. We find that fasting glucose is higher, with a trend for higher cholesterol, in BCS compared to non-cancer controls. We also determined that BCS are more likely to be treated for depression than non-cancer controls and that BCS with depression have more metabolic abnormalities. Thus, understanding the consequences of breast cancer diagnosis and treatment on risk for depression and metabolic dysfunction may aid in medical monitoring to optimize health during the survivorship phase of cancer recovery.

The pathophysiological response linking depression and metabolic dysfunction is inconclusive with regard to causal factors. It is postulated that depression is associated with increased stress and activation of the hypothalamic–pituitary–adrenal (HPA) axis, which is involved in the pathogenesis of central adiposity and metabolic syndrome [25]. Greater metabolic dysfunction is associated with higher proinflammatory cytokines [26], many of which are elevated in BCS [27, 28]. Conversely, there is evidence that many cancer treatments, including radiation and chemotherapy may activate an immune response [29, 30], leading to dysregulation of the HPA axis and, ultimately, depression [31]. Further, development of central adiposity also increases secretion of endogenous steroid hormones in obese, postmenopausal women [32]. High levels of circulating estrogen and testosterone are associated with an increased risk of breast cancer in peri- and postmenopausal women [33, 34]. Although, we do not believe estrogen concentrations have been compared between BCS and non-cancer controls, no differences in hyperandrogenic status (testosterone concentrations >1.2 ng/ml) were found between these groups previously, despite observed metabolic differences [15]. Studies, including ours, suggest that as many as 50–55 % of BCS are depressed [35–37], which is ~20–25 % more than that observed in postmenopausal women without a history of cancer [38]. Further, our results agree with previous reports [14, 39] that metabolic syndrome is prevalent in ~50 % of postmenopausal BCS. This is ~1.5 higher than what is observed in postmenopausal women of comparable age and BMI in NHANES [40] and our postmenopausal non-cancer controls. The presence of metabolic abnormalities and depression following breast cancer diagnosis are associated with elevated cancer recurrence and mortality rates [39, 41, 42], highlighting the importance of understanding the mechanism linking depression and metabolic dysfunction in BCS.

Often the failure to recover after breast cancer treatment (i.e. low quality of life persisting for years following completion of cancer treatment [43]) is attributed to lifestyle choices, including low activity levels and suboptimal nutrient intake. However, our results suggest that the metabolic abnormalities and depression observed in BCS may not be attributable to low cardiorespiratory fitness, as these risk factors were present even though a higher VO_2_max was observed in BCS than non-cancer controls. Despite this finding, many studies suggest that depressive symptoms and metabolic dysfunction improve with exercise in BCS [44–46].

This study is limited by a small sample size, which prevented stratification by breast cancer treatments and the control of length of time post-cancer treatment. As there is evidence that breast cancer treatments, including chemical variations in drugs used for chemotherapy, may have varying effects on metabolism [47], future studies should attempt to stratify by treatment. There is limited data available on the impact of duration of survivorship on the observed relationships; however, there is evidence that subjective outcomes (i.e. quality of life and depression) remain lower than pre-diagnosis or may even continue to decline during the survivorship phase [3, 48]. The cause of this continued failure to recover is not elucidated and may be influenced by factors other than the cancer diagnosis and treatment (i.e. financial and emotional support) during the survivorship phase. Although the gold standard for depression evaluation is the use of a clinician rated interview technique, such as the Structured Clinical Interview for DSM-IV Axis I Disorders and the Hamilton Depression Rating Scale, these methods require substantial subject time and trained clinical interviewers [49]. Self-report questionnaires, such as the CES-D, have strong validity and reliability [50] and are less burdensome [51] when compared to interview techniques. Further, because of the pilot nature of this study, body composition assessments of the BCS were limited to anthropometric measurements. While more sophisticated measures of body composition may provide additional insight as to the role of cancer survivorship on metabolism, these tests can be expensive and often require radiation exposure, which may decrease participant interest in and compliance to the study protocol. However, some of these limitations are balanced by our strong study design, where BCS were matched for age, race, and BMI to non-cancer controls. BMI of the subjects spanned from normal weight to morbidly obese, while most studies examining metabolic dysfunction in BCS only includes those who are obese; thus, these findings may be more representative of the general BCS population.

In summary, our results support the need to monitor weight gain, depression, and the progression of metabolic abnormalities during treatment and longitudinally in BCS, as their development may affect long-term survivorship. Further studies into the mechanistic links between depression and metabolism are necessary to identify strategies that can offset their impact on survivorship following a breast cancer diagnosis.
